# Brain Activation in Motor Sequence Learning Is Related to the Level of Native Cortical Excitability

**DOI:** 10.1371/journal.pone.0061863

**Published:** 2013-04-16

**Authors:** Silke Lissek, Guido S. Vallana, Onur Güntürkün, Hubert Dinse, Martin Tegenthoff

**Affiliations:** 1 Department of Neurology, BG-Kliniken Bergmannsheil, Ruhr-University Bochum, Bochum, Germany; 2 Institute of Cognitive Neuroscience, Department Biopsychology, Faculty of Psychology, Ruhr-University Bochum, Bochum, Germany; 3 Neural Plasticity Lab, Institute for Neuroinformatics, Ruhr-University Bochum, Bochum, Germany; University Of Cambridge, United Kingdom

## Abstract

Cortical excitability may be subject to changes through training and learning. Motor training can increase cortical excitability in motor cortex, and facilitation of motor cortical excitability has been shown to be positively correlated with improvements in performance in simple motor tasks. Thus cortical excitability may tentatively be considered as a marker of learning and use-dependent plasticity. Previous studies focused on changes in cortical excitability brought about by learning processes, however, the relation between native levels of cortical excitability on the one hand and brain activation and behavioral parameters on the other is as yet unknown. In the present study we investigated the role of differential native motor cortical excitability for learning a motor sequencing task with regard to post-training changes in excitability, behavioral performance and involvement of brain regions. Our motor task required our participants to reproduce and improvise over a pre-learned motor sequence. Over both task conditions, participants with low cortical excitability (CElo) showed significantly higher BOLD activation in task-relevant brain regions than participants with high cortical excitability (CEhi). In contrast, CElo and CEhi groups did not exhibit differences in percentage of correct responses and improvisation level. Moreover, cortical excitability did not change significantly after learning and training in either group, with the exception of a significant decrease in facilitatory excitability in the CEhi group. The present data suggest that the native, unmanipulated level of cortical excitability is related to brain activation intensity, but not to performance quality. The higher BOLD mean signal intensity during the motor task might reflect a compensatory mechanism in CElo participants.

## Introduction

Task training and learning can induce changes in the brain - not only in cortical representations [1], [2] but also in cortical excitability. For a variety of tasks, studies have demonstrated increases in cortical excitability after training: Motor cortex excitability was shown to increase during execution and mental imagery of sequential finger movements, but not during repetitive movements [3]. However, another study demonstrated that also simple repetitive movements such as ballistic abductions of the thumb caused an increase in MEP amplitude indicative of training effects [4], [5]. Extensive training of elementary finger tapping movements also increased cortical excitability [6]. Moreover, the increase in cortical excitability appears to be related to learning success: task-induced facilitation of motor evoked potentials (MEPs) was found to be positively correlated with improvements in performance [7], [8]. Since primary motor cortex does not only control muscle activity and movements, but appears to be involved in forming new or adapting existing motor skills [9], as required in practicing of learned movements [10], [11] or in learning of a new movement sequence [12], changes in cortical excitability in motor cortex might be considered a marker for motor learning and use-dependent plasticity.

Investigations on cortical excitability primarily focused on the effects of training upon the level of cortical excitability, without considering a potential interindividual baseline variability. However, if cortical excitability is a marker of learning and use-dependent plasticity, the pre-existent interindividual variability of native cortical excitability in itself may be an – as yet unresearched – contributor and modulator of the subsequent learning success, and may also reflect in the respective brain activation patterns.

Cortical excitability can be reliably assessed by paired-pulse transcranial magnetic stimulation (TMS) techniques, which deliver a sequence of two magnetic stimuli of sub-threshold and supra-threshold intensity separated by a variable interstimulus interval. This method measures the suppression or enhancement of the second stimulus by the first and thus provides information about cortical inhibition and facilitation [13], [14], with short ISIs around 2–4 ms providing inhibition and longer ISIs around 10–15 ms providing facilitation, respectively [8]. There is evidence that this kind of paired-pulse stimulation yields measures of intracortical or cortico-cortical excitability on the level of primary motor cortex [13], [14]. These intracortical facilitation (ICF) and short-latency intracortical inhibition (ICI) phenomena are assumed to be mediated by glutamate and GABA, respectively [14], [15]. The N-methyl-D-aspartate (NMDA) antagonist memantine can enhance intracortical inhibition and reduce intracortical facilitation, indicating NMDA receptor involvement in the regulation of cortical excitability [16].

Imaging studies on motor sequence processing mostly test participants with prelearned sequences that are performed either self-paced [17]–[19] or paced by an external clock [20], [21]. Such tasks require repeating the same motor pattern over and over, e.g. they ask for low variability of motor behavior. High variability of motor output, in contrast, occurs when deliberately varying a prelearned pattern, or in musical improvisation, and has been researched in studies on musicians, particularly on piano players [22]–[24].

Since we were interested in investigating the relation between cortical excitability and brain activation under these distinct demands, we designed a motor task that contained the conditions of a) reproduction of a learned motor sequence and of b) improvisation over the learned motor sequence, with both conditions posing comparable demands upon motor activity, i.e. work load and involvement of effectors (hand and fingers), requiring participants to exhibit both low and high variability of motor behavior. In parallel, we investigated whether differential native cortical excitability had an effect upon learning performance and improvisation behavior.

If motor cortical excitability is a general marker for use-dependent plasticity, as suggested by a learning-related increase observed in several studies, a post-training increase in cortical excitability should occur regardless of differential baseline levels. Moreover, if native cortical excitability is an indicator of learning performance, corresponding to results from studies reporting increases in post-training cortical excitability to signal learning, then participants with a higher native level of motor cortical excitability should show superior motor learning performance.

## Materials and Methods

### Participants

18 healthy volunteers (11 females, 7 males), mean age 25.66 years, range 23–34 years, st.dev. 3.3254 years, without a history of neurological disorders participated in this study. All subjects were right-handed as measured by the Edinburgh Handedness Inventory (Oldfield, 1971), with a mean laterality index of +74.13 (SD = 18.37, ranging from +25 to +100). Participants received a monetary compensation for their participation (in the amount of € 100).

### Ethics Statement

All subjects participated in this study after giving written informed consent. The protocol was approved by the local ethics committee of the Ruhr-University Bochum. The study conforms to the Code of Ethics of the World Medical Association (Declaration of Helsinki). Prior to the experiments, participants received handouts informing them about the TMS and MRI procedures and the instructions for the motor sequencing task.

### Motor sequence task

**Figure 1:**
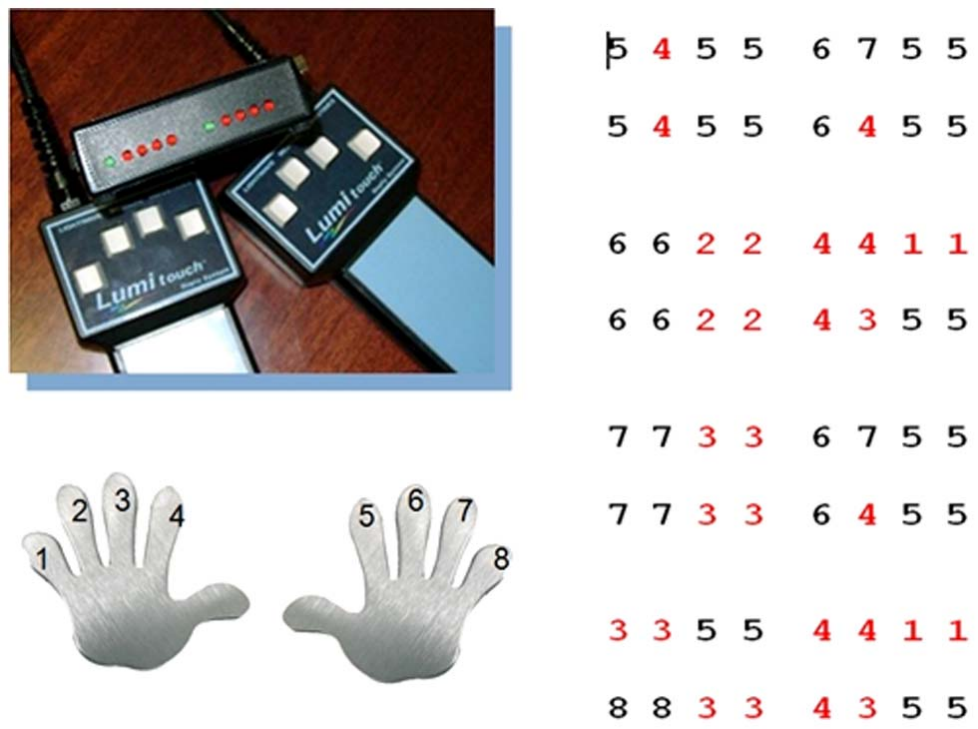
Motor sequence task. Participants learned a motor sequence consisting of a series of key presses on two keyboards with each four keys for the left and the right hand (red: left hand, black: right hand). Each digit corresponded to a key on the keyboard and thus to a finger in ascending order from left to right, with 1 corresponding to the left and 8 to the right pinky finger. The motor sequence consisted of 64 key presses in total, subdivided into 4 sections with 16 key presses each. In total there were 9 different 4-key sequences.

To learn and perform the motor sequencing task, participants underwent two scanning sessions in the MRT-scanner. The first scanning session was a training session, during which participants learned the motor sequencing task in an interactive training paradigm. They were taught to perform a series of prescribed finger tapping sequences on two fMRI-ready keyboards (LUMItouch response pads, Photon Control Inc., Canada) with four keys each, using the index, middle, ring and pinky fingers of both hands. Each key was associated with a certain sound (sound files of guitar chords). In total, there were 4 motor sequences with 16 key presses each to be learned (requiring 64 key presses in total), containing 9 different 4-key blocks. Each tapping sequence was defined as a series of digits describing the keys and thus the fingers of the left and the right hand in ascending order, with digit 1 corresponding to the left pinky finger, 4 and 5 corresponding to left and right index finger, respectively, and 8 corresponding to the pinky finger of the right hand (see Fig. 1). A sequence such as “4 3 5 5” thus requires the following series of key presses: left index finger, left middle finger and two times right index finger. During learning, the prompts and the motor sequences in question were displayed via fMRI-ready LCD-goggles and the sounds were displayed via fMRI-ready headphones (both: Visuastim Digital, Resonance Technology Inc., Northridge, CA, USA). In the first part of the learning session, participants were shown partial sequences of 16 key presses each, while the associated sound sequences were played to them over the headphones. They were then requested to play the sequence themselves, aided by the visual display of the key sequence on the screen. Feedback was given regarding the percentage of correct responses, with 80% correct responses defining the sequence as learned. Once a sequence was defined as learned under these conditions, the process was repeated, but this time without the visual prompts, requiring participants to play the sequence by rote. Again feedback was given regarding the percentage of correct responses. Once all single sequences were successfully played by rote, the process was repeated with the complete motor sequence (64 key presses). After successful completion of this final training part, the training stopped. For participants who did not succeed in achieving the learning criterion of 80 %, the maximum duration of the training was set to about 18 minutes (340 recorded volumes at a TR of 3200 ms).

Prior to the learning session in the MRT scanner, participants were given the opportunity to make themselves familiar with the motor sequences on the keyboards. They were instructed to memorize the sequences and to train the succession of key presses on a life-size printout of the keyboards used in the MRT scanner.

The second scanning session was the experimental session which evaluated the actual result of the training session. We used a block design with three conditions: simple finger tapping as a rest phase, a Performance condition to evaluate the correctness of reproduction of the learned motor sequence (condition of low variability), and Improvisation condition (condition of high variability). In the Tapping condition, participants were requested to alternately press the keys of the right and left index finger. In the Performance condition, participants were requested to perform the complete motor sequence they had learned during the training. In the Improvisation condition, participants were asked to alter the sequences to their liking while still maintaining a resemblance to the learned sequences. The three conditions were presented in alternating blocks, 7 per condition, resulting in 7×34 = 238 recorded volumes. Each block of Tapping lasted 25,6 seconds (8 volumes); each block of Performance and Improvisation lasted 41,6 seconds (13 volumes), respectively, in each case including an instruction prompt that was presented for 3,2 seconds (1 volume) announcing the following block. The complete experimental session had a fixed duration of approx. 13 minutes (238 recorded volumes).

### Determination of cortical excitability by means of TMS and MEPs

Before and after the fMRI session, motor cortical excitability in terms of intracortical inhibition and facilitation of the participants was determined by means of single- and double-pulse TMS over primary motor cortex. Magnetic pulses were delivered using a Bistim module connected to two Magstim Rapid 200 (Magstim Co., Whitland, Wales, United Kingdom) stimulators. The stimuli were applied via a flat circular coil (outer diameter 14 cm) centered over the vertex (Cz) with the current flowing counterclockwise in the coil to activate predominantly the left hemisphere, and clockwise to activate predominantly the right hemisphere. While stimulating the contralateral hemisphere, MEPs were recorded by means of Ag-AgCl surface electrodes positioned on the target muscle, the first dorsal interosseus (FDI) muscle of the right hand.

The resting motor threshold was defined as the minimum stimulus intensity at which 4 out of 8 stimuli evoked an MEP with an amplitude of >50 µV in the relaxed FDI muscle, expressed in percent of maximum stimulator output. To determine the threshold, stimulus intensity was increased in increments of 5 % from a subthreshold level, until threshold and above threshold responses were achieved, and then the stimulus intensity was adjusted in order to conform to the above mentioned criteria.

Stimulation intensity for single pulses was 120% of the motor threshold thus determined, for double pulses 80 % and 120 %. A series of 8 stimulations each was delivered for single-pulse stimulation and double-pulse stimulation with 2 ms, 10 ms, 4 ms and 15 ms ISI. Area and peak-to-peak amplitude of the MEP response were recorded and stored for off-line analysis by means of a NEUROPACK M1 MEB 9200 EVP/EMG measuring system (Nihon Kohden, Japan).

### Analysis of cortical excitability data

From the data acquired according to the procedure described above, amplitude ratios of cortical excitability were calculated by dividing the mean amplitude of the respective double-pulse stimulation series by the mean amplitude of the two sets of single-pulse stimulation series (Kujirai et al., 1993; Schwenkreis et al., 1999, 2003). The ratios were calculated separately for ISIs with inhibitory effects (2 and 4 ms) and ISIs with facilitatory effects (10 and 15 ms). These amplitude ratios reflect the degree to which a facilitatory or inhibitory double-pulse stimulation evokes a higher (values>1) or lower (values<1) response than the control single-pulse stimulation.

To investigate the role of cortical excitability for motor task performance and improvisation, we assigned participants to one of two groups - high or low cortical excitability. To do this, we calculated the median for each of four cortical excitability measures: amplitude ratios of mean facilitatory double pulse stimulation (ISI 10 and 15 msec) and of mean inhibitory double pulse stimulation (ISI 4 and 2 ms), each pre and post training. For each data set, each participant received a rating reflecting whether his individual value was above or below the median: h for high cortical excitability, i.e. above the median, and l for low cortical excitability, i.e. below the median, resulting in a total of four ratings. Participants with ratings of 4× h (n = 0) or 3× h (n = 8) were assigned to the high cortical excitability group (CEhi), while subjects with ratings of 4× l (n = 5) or 3× l (n = 3) were assigned to the low cortical excitability group (CElo). The remaining subjects with a combination of 2× h and 2× l (n = 2) were assigned to the groups according to their pre-training results 2× l (n = 1) was included in CElo, and 1× l 1× h (n = 1) was included in CEhi, resulting in each group consisting of n = 9 participants.

In all cases the assignment to a group would have been identical if it were based on pre-training values only. Thus, the assignment reflects both the native level of CE pre training and the overall CE level pre and post training. The distribution of male and female participants was as follows: of 7 male participants, 3 were assigned to CEhi and 4 to CElo. Of the 11 female participants, 6 were assigned to CEhi and 5 to CElo.

### Imaging data acquisition

Images were acquired using a whole-body 3T scanner (Philips Achieva 3.0 T X-Series, Philips, The Netherlands) with a 32-channel SENSE head coil.

Blood-oxygen level dependent (BOLD) contrast images were obtained with a dynamic T2* weighted gradient echo EPI sequence using SENSE (TR 3200 ms, TE 35 ms, field of view 224 mm, slice thickness 3.0 mm, voxel size 2.0×2.0×3.0 mm). We acquired 45 transaxial slices parallel to the anterior commissure — posterior commissure (AC-PC) line which covered the whole brain. Two imaging sessions were conducted, the first (maximum 340 volumes) for training of the motor sequence, the second (238 volumes) for the actual experimental task.

Additionally, anatomical images of each subject were acquired using an isotropic T1 TFE sequence (field of view 240 mm, slice thickness 1.0 mm, voxel size 1×1×1 mm) with 220 transversally oriented slices covering the whole brain.

### Imaging data analysis

For preprocessing and statistical analysis of the fMRI data, we used the Statistical Parametric Mapping (SPM) Software, Version 8 (Wellcome Department of Cognitive Neurology, London, UK) implemented in Matlab (Mathworks, Sherbon, MA). The first 3 images of each fMRI session (total 340 resp. 238 images), during which the BOLD signal reaches steady state, were discarded from further analysis to remove non-steady state effects caused by T1 saturation. To correct for between-scan movements, all volumes were realigned to the first volume. Functional images were spatially normalized into standard stereotactic coordinates at 2×2×2 mm^3^ using an EPI template provided by the Montreal Neurological Institute (MNI). Smoothing was conducted with a 6 mm full-width half-maximum (FWHM) kernel, in accordance with the standard SPM procedure. The acceptable limit for head motion was 2 mm for translational movements and 0.5° for rotational movements.

In a first-level single-subject analysis, changes in the BOLD response for each participant were assessed by linear combinations of the estimated GLM parameters which are displayed by the individual contrast images. This analysis was performed by modeling the Performance and Improvisation conditions as explanatory variables convolved with a standard hemodynamic response function as implemented in SPM 8. Contrast images were calculated that compared activation in two contrasts: Improvisation>Performance and Performance>Improvisation, in order to differentiate regions that are particularly active during either low or high variability of behavior.

In a second-level random-effects analysis, the individual contrast images of members of both groups were entered into two one-sample t-tests to display the commonly activated regions of all 18 participants for the contrasts Improvisation>Performance and Performance>Improvisation. The resulting statistical parametrical map was thresholded at p<0.0001 and p<0.001 uncorrected and a minimum cluster size of k = 10. For the clusters found activated in the above mentioned contrasts, we extracted mean signal intensities of activation for each participant by means of the corresponding function in the Marseille Region of Interest Toolbox for SPM MarsBaR [25]. Further statistical analyses comparing mean signal intensities of the two groups for the activated regions by means of ANOVA and t-tests for independent samples were performed using the SPSS 20 software package (IBM SPSS Statistics for Windows, Version 20.0, Armon, NY: IBM Corp.).

### Behavioral data acquisition

Each key press of the participants during the three conditions was recorded and stored by means of the motor sequence task software in log files with a format that could be read out in statistics and spreadsheet software.

### Behavioral data analysis

Behavioral data were analyzed with regard to correctness in the Performance condition and with regard to the level of improvisation in the Improvisation condition.

Correctness was evaluated as adherence to the prescribed motor sequence. The percentage of correct keystrokes was determined by comparing the participant's motor sequence with the prescribed motor sequence. Speed was not taken into consideration, i.e. if a participant did not manage to go through the complete motor sequence within one block, then his executed sequence was compared to the prescribed motor sequence of the same length. Deviations from the prescribed motor sequence, i.e. tapping keys in a different order than stipulated in the sequence, were considered errors. However, the evaluation procedure took into account errors that were obviously corrected. If for example the participant started a wrong part of the sequence and realized and corrected that error after maximal four keystrokes by continuing with the correct sequence, the wrong keystrokes were cut from the total sequence. The analysis yielded values for percentage correct for each repetition block of the task (total 7) and the mean percentage correct value for the complete 7 repetitions/blocks. To further account for initial problems or intermediate lapses in attention, for the final analysis, only the 5 best repetitions/blocks of each participant were taken into consideration.

The level of improvisation in the Improvisation condition was determined by computing the Shannon-Wiener information entropy [26] – a measure of the randomness of a probability distribution of values which is often used to measure diversity in categorical data. The entropy was computed for each participant, based on the amount of preserved strings of minimum 4 key presses that still corresponded to the prescribed motor sequence, irrespective of their position of the improvised sequence performed by the participant. In this analysis, large entropy values indicated low randomness, i.e. strong adherence to the prelearned motor sequence, while low entropy values indicated high randomness, i.e. a high level of deviation from the prelearned sequence. High levels of improvisation thus reflect in low index values and vice versa.

## Results

### Cortical excitability results

An F-test for repeated measures showed a significant main effect of group for both facilitatory (F(1,17) = 9.795 p = .006) and inhibitory (F(1,17) = 10.919 p = .004) stimulation ratios, while the main effect of time of measurement was not significant, neither for facilitatory (F(1,17) = 1.566 p = .229) nor for inhibitory (F(1,17) = .468 p = .504) stimulation ratios. For facilitatory stimulation ratios, a significant group*time of measurement interaction was found (F(1,17) = 5.181 p = .037), while the same interaction was not significant for inhibitory stimulation ratios (F(1,17) = 3.776 p = .070).

#### Differences between groups CEhi and CElo

**Figure 2:**
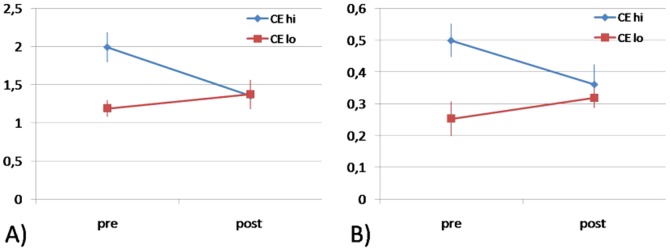
Cortical excitability in CEhi and CElo participants pre and post learning and training of the motor sequence task: mean CE expressed as the ratio of MEP amplitudes in response to A) facilitatory and B) inhibitory double pulse/single pulse stimulation pre and post training.

Mean amplitude ratios of cortical excitability *pre* training differed significantly between the two groups (t-test for independent samples): facilitatory stimulation ratio t(16) = 3.553 p = .004 (means CEhi 1.9943 +/− s.e.m. 0.1959, CElo 1.1897 +/− s.e.m. 0.1136), inhibitory stimulation ratio t(16) = 4.385 p = .002 (means CEhi 0.4995 +/− s.e.m. 0.0545; CElo 0.2531 +/− s.e.m. 0.0137). On the other hand, means of cortical excitability *post* training did not show significant group differences: facilitatory stimulation ratio t(16) = −0,103 p = .920 (means CEhi 1.3537 +/− s.e.m. 0.0936; CElo 1.3758 +/− s.e.m. 0.1927) and inhibitory stimulation ratio t(16) = 0.538 p = .601 (means CEhi 0.3615 +/− s.e.m. 0.0702; means CElo 0.3192 +/− s.e.m. 0.0353), indicating that cortical excitability levels of the two groups tended to converge to similar levels post training, as shown in Figure 2.

#### Differences of CE between pre and post measurements within groups CEhi and CElo

While the CEhi group showed a significant decrease of the CE ratio post compared to pre training in response to facilitatory stimulation t(8) = 2.451 p = .040, the difference in inhibitory stimulation was not significant t(8) = 1.448 p = .186, indicating significantly reduced facilitatory CE, and a slightly, albeit insignificantly, increased inhibitory CE (lower inhibitory CE values indicate higher inhibition).

In the CElo group, neither difference between pre and post values was significant, despite a slight tendency towards higher facilitation and less inhibition. (facilitatory t(9) = −0.738 p = .482; inhibitory t(9) = −1.497 p = .173).

### Behavioral results

#### Correctness of performance

The complete group of participants (n = 18) achieved an average of 76.94 % (+/−5.80 standard error of means) correct key presses. CEhi participants achieved a mean value of 77.58 % (+/−8.89 s.e.m.) and CElo of 76.29 % (+/−7.98 s.e.m.), the group difference is not significant (t(16) = 0.107 p = .916).

#### Degree of improvisation

The Shannon-Wiener Index is used to measure diversity in categorical data, with high diversity being reflected in low values and low diversity being reflected in high values of the index.

The Shannon-Wiener Index of the complete group of participants shows a mean value of 1.1805 (+/− s.e.m. 0.1268). CEhi subjects achieve a mean of 1.2894 (+/− s.e.m 0.1499) and CElo subjects of 1.0716 (+/−s.e.m. 0.2071). The group difference is not significant (t(16) = .852 p = .407).

In sum, the results indicate that cortical excitability per se does not appear to have a significant influence upon the quality of the performance in the motor sequencing task.

### Imaging results

#### Activation differences between Improvisation and Performance (n = 18)

**Figure 3:**
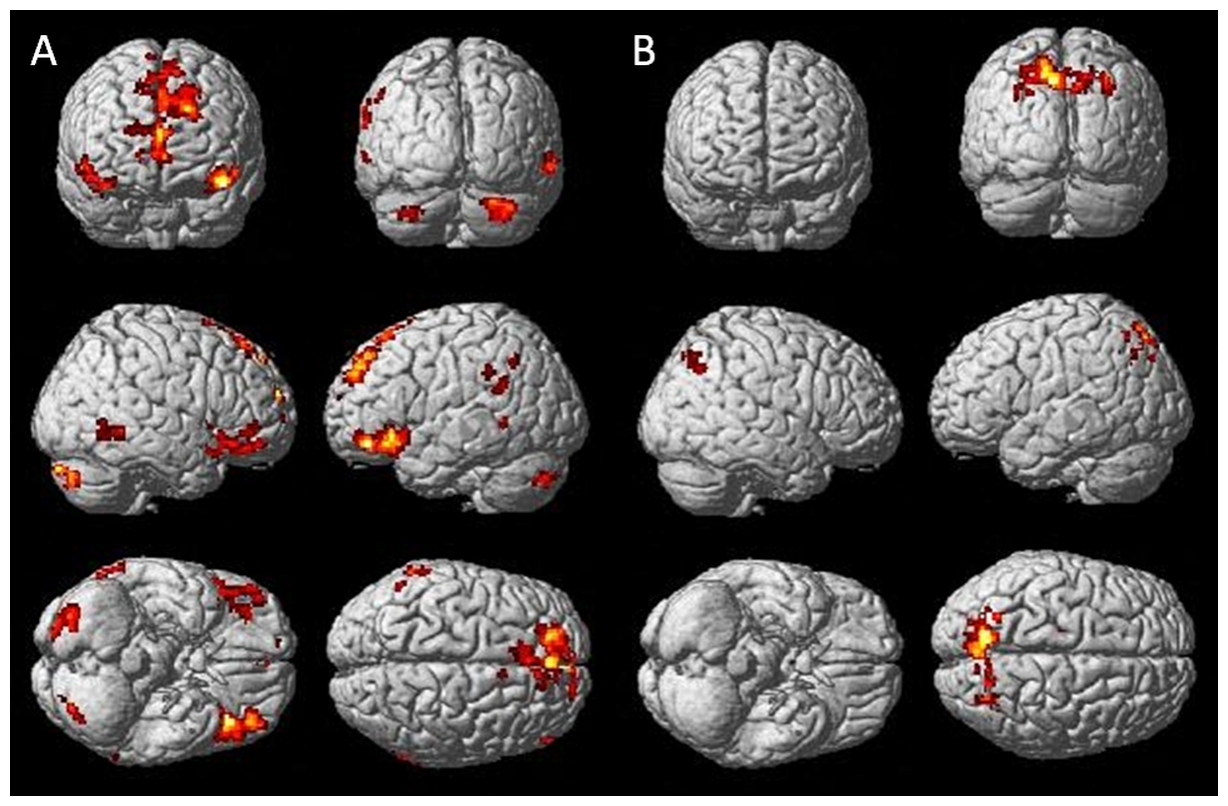
Brain activation in the complete sample (n = 18) during the contrasts A) improvisation>performance (t = 4.71 p<0.0001) and B) performance>improvisation (t = 3.65 p<0.001). During improvisation of the motor sequence, regions in bilateral orbitofrontal cortex (BA 10, 11, 47), dorsolateral PFC (BA 8, 9), anterior cingulate (BA 32) middle temporal gyrus (BA 21), left supramarginal gyrus and inferior parietal lobule were activated. During performance of the learned motor sequence, higher activation was observed in bilateral precuneus (BA 7, 31), posterior cingulate/retrosplenial cortex regions (BA 30, 31), insula, caudate tail and body and in left prefrontal cortex (BA 6, 8).

During improvisation of the motor sequence task, compared to performance (one-sample t-test, height threshold t = 4.71, p<0.0001 uncorrected, extent threshold k = 10 voxels) for the complete sample (n = 18) we found higher activation in bilateral orbitofrontal cortex (BA 10, 11,47), bilateral dorsolateral PFC (BA 8,9), bilateral anterior cingulate (BA 32), bilateral middle temporal gyrus (BA 21), left supramarginal gyrus and inferior parietal lobule (BA 40) (see Figure 3a and Table 1).

**Table 1:** Activated areas in the contrast Improvisation>Performance for the complete sample (n = 18), one-sample t-test, height threshold t = 4.71 p<0.0001, extent threshold k = 10 voxels.

Brain Area	BA		MNI coordinates			t-value	cluster
			X	Y	Z		
**Orbitofrontal cortex**							
**Middle frontal gyrus**	**11**	**L**	**−38**	**42**	**−14**	**10.46**	**657**
		**R**	**46**	**48**	**−14**	**7.67**	**348**
			**32**	**42**	**−14**	**5.40**	**In 348**
**Inferior frontal gyrus**	**47**	**L**	**−40**	**28**	**−14**	**8.42**	**In 657**
			**−32**	**18**	**−15**	**5.37**	**In 657**
		**R**	**48**	**30**	**−12**	**5.10**	**In 348**
**Medial frontal gyrus**	**10**	**L**	**−4**	**60**	**15**	**7.06**	**In 1327**
		**R**	**4**	**50**	**4**	**5.99**	**In 1327**
			**12**	**60**	**0**	**6.61**	**21**
**Dorsolateral prefrontal cortex**							
**Superior frontal gyrus**	**9**	**L**	**−20**	**48**	**34**	**8.49**	**1327**
			**−22**	**44**	**38**	**5.32**	**In 1327**
	**8**	**L**	**−16**	**40**	**48**	**7.02**	**In 1327**
			**−22**	**40**	**45**	**5.55**	**In 1327**
		**R**	**6**	**36**	**54**	**5.49**	**In 1327**
			**10**	**42**	**52**	**5.45**	**In 1327**
**Cingulate**							
**Anterior cingulate**	**32**	**R**	**6**	**45**	**4**	**7.33**	**In 1327**
		**L**	**−10**	**46**	**2**	**6.04**	**15**
**Temporal**							
**Supramarginal gyrus**	**40**	**L**	**−64**	**−50**	**24**	**6.86**	**48**
**Middle temporal gyrus**	**21**	**R**	**64**	**−50**	**−4**	**6.84**	**106**
			**62**	**−60**	**0**	**5.10**	**In 106**
		**L**	**−64**	**−52**	**2**	**5.78**	**23**
**Inferior temporal gyrus**	**37**	**R**	**60 -**	**−60**	**−10**	**5.50**	**In 106**
**Parietal**							
**Inferior parietal lobule**	**40**	**L**	**−54**	**−56**	**40**	**6.27**	**26**
			**−62**	**−40**	**36**	**5.90**	**25**
**Cerebellum**							
**Posterior lobe, declive**		**R**	**28**	**−90**	**−30**	**8.78**	**291**
**Posterior lobe, pyramis**		**R**	**24**	**−78**	**−38**	**5.55**	**In 291**
**Posterior lobe, uvula**		**R**	**35**	**−80**	**−34**	**5.53**	**In 291**
		**L**	**−24**	**−86**	**−35**	**5.58**	**In 83**
**Posterior lobe, tuber**		**L**	**−30**	**−78**	**−40**	**6.57**	**83**
			**−38**	**−74**	**−38**	**5.73**	**In 83**

#### Group differences in mean signal intensity in brain regions activated in contrast improvisation>performance

To compare brain activation levels of CElo and CEhi participants during improvisation, we derived mean signal intensities from commonly activated brain regions in the above contrast (one-sample t-test, n = 18, height threshold t = 4.71 p<0.0001 uncorrected, extent threshold k = 10 voxels).

An ANOVA with the between-subject factor ‘group’ and the within-subjects factor ‘region’ showed a significant main effect of group F(1,16) = 4.643, p = .047, indicating that the CElo group showed overall significantly higher mean signal intensities across the regions active during improvisation than the CEhi group. The main effect of area was also significant, F(11,176) = 4.222 p = .000, the interaction area*group was not significant F(11,176) = 1.490 p = .406.

T-tests for independent samples (two-tailed) showed significant differences between CEhi and CElo in bilateral orbitofrontal cortex (BA 11/47) (t(16) = 2.188, p = .044, in left anterior cingulate (BA 32) and regions in dorsolateral PFC (BA 9, 8) (t(16) = 2.464, p = .025), and in left BA 40 (t(16) = −2.759, p = .014). In all of these regions, CEhi subjects show significantly less activation than CElo subjects.

**Figure 4:**
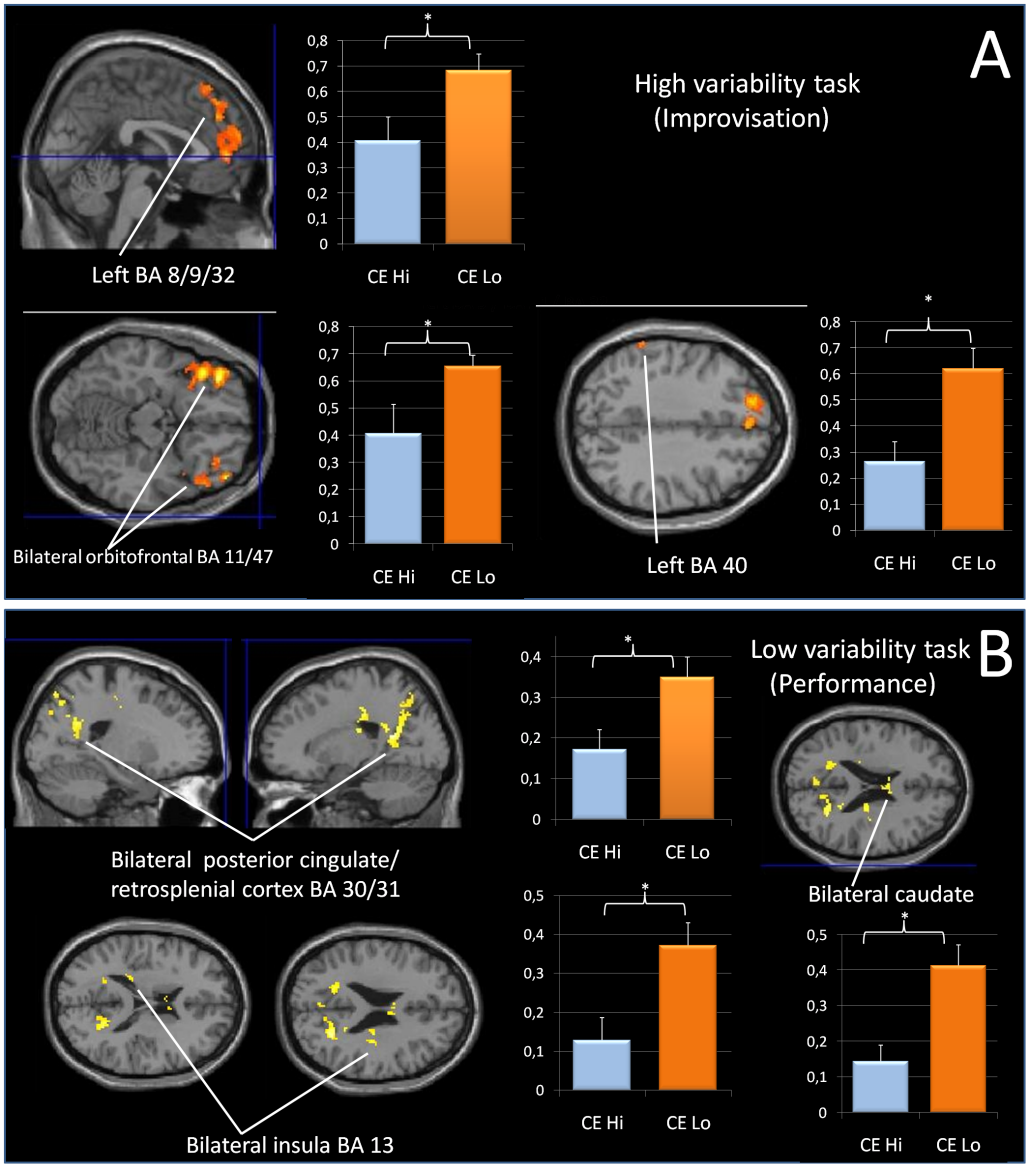
Differences in BOLD mean signal intensity between CEhi and CElo groups during improvisation and reproduction (performance) of the motor sequence task. A) Regions that show significant differences between CEhi and CElo groups during the improvisation condition. B) Examples of regions that show significant differences between CEhi and CElo groups during the reproduction of the motor sequence task.

See Fig. 4a) and Table 2 for a complete list of individual regions which showed significant differences between mean signal intensities of CEhi and CElo groups.

**Table 2:** Brain areas in which CElo group activation is significantly higher than CEhi group activation during improvisation over the motor sequence task.

Brain Area (BA)	MNI peak coordinate	Cluster size	t-value	p-value
**Mean right anterior cingulate (BA 32)/superior frontal gyrus (BA 9)/medial frontal gyrus**	−20 48 34/6 45 4/−5 45 40	1327	t(16) = 2.464	p = .025
**Right anterior cingulate (BA 32)**	6 45 4	92	t(16) = 2.76	p = .015
**Left superior frontal gyrus (BA 9)**	−20 48 34	327	t(16) = 2.44	p = .049
**Left medial frontal gyrus (BA 8)**	−5 45 40	228	t(16) = 2.285	p = .037
**Mean bilateral orbitofrontal cortex (BA 11/47)**	−38 42 −14/46 48 −14	1015	t(16) = 2.188	p = .044
**Left OFC (BA 11/47)**	−38 42 −14	657	t(16) = 2.157	p = .049
**Right OFC (BA 11/47)**	46 48 −14	348	t(16) = 2.597	p = .023
**Left supramarginal gyrus (BA 40)**	−64 −50 24	48	t(16) = 2.759	p = .014

#### Activation differences between Performance and Improvisation (n = 18)

During performance of the motor sequence task, compared to improvisation (one-sample t-test, height threshold t = 3.65, p<0.001 uncorrected, extent threshold k = 10 voxels), the complete group (n = 18) showed higher activation in bilateral precuneus (BA 7,31), in cingulate regions (right posterior cingulate/retrosplenial cortex BA 30 and bilateral cingulate gyrus BA 31), bilateral insula (BA 13), bilateral caudate tail and body and in clusters in left prefrontal cortex (BA 6, 8) (see Figure 3b and Table 3).

**Table 3:** Activated areas in the contrast Performance>Improvisation for the complete sample (n = 18), one-sample t-test, height threshold t = 3.65 p<0.001, extent threshold k = 10 voxels.

Brain Area	BA		MNI coordinates			t-value	cluster
			X	Y	z		
**Prefrontal cortex**							
**Middle frontal gyrus**	**8**	**L**	**−28**	**12**	**38**	**4.72**	**20**
	**6**	**L**	**−22**	**−12**	**44**	**4.13**	**11**
**Precentral gyrus**	**6**	**L**	**−20**	**−18**	**50**	**4.34**	**12**
**Caudate**							
**Caudate tail**		**R**	**30**	**−36**	**2**	**5.99**	**36**
		**L**	**−32**	**−40**	**4**	**4.46**	**17**
**Caudate body**		**L**	**−5**	**4**	**22**	**4.04**	**In37**
		**R**	**4**	**4**	**22**	**4.86**	**37**
			**22**	**−20**	**22**	**4.43**	**26**
			**4**	**8**	**16**	**4.22**	**10**
**Cingulate**							
**Posterior cingulate/retrosplenial cortex**	**30**	**R**	**20**	**−54**	**16**	**5.55**	**1412**
**Cingulate gyrus**	**31**	**L**	**−20**	**−56**	**25**	**4.93**	**In 1412**
		**R**	**20**	**−26**	**36**	**4.87**	**34**
			**20**	**−46**	**22**	**4.56**	**13**
**Parietal**							
**Precuneus**	**7**	**L**	**−2**	**−75**	**48**	**5.33**	**In 1412**
			**−18**	**−64**	**34**	**5.27**	**In 1412**
			**−8**	**−70**	**46**	**4.82**	**In 1412**
			**−12**	**−74**	**52**	**4.73**	**In 1412**
			**−24**	**−62**	**52**	**4.49**	**54**
			**−24**	**−80**	**40**	**4.04**	**16**
		**R**	**14**	**−65**	**45**	**4.75**	**In 1412**
			**12**	**−70**	**42**	**4.49**	**In 1412**
	**31**	**L**	**−4**	**−70**	**24**	**3.99**	**16**
**Angular gyrus**	**39**	**R**	**34**	**−62**	**35**	**4.98**	**In 1412**
**Inferior parietal lobule**	**40**	**L**	**−34**	**−58**	**42**	**3.77**	**In 54**
**Insula**	**13**	**R**	**34**	**−20**	**24**	**4.48**	**15**
		**L**	**−26**	**−32**	**16**	**4.31**	**15**

#### Group differences in mean signal intensity in brain regions activated in contrast performance>improvisation

To compare brain activation levels of the groups during performance, we derived mean signal intensities from commonly activated brain regions shown in the above contrast.

An ANOVA with the between-subject factor ‘group’ and the within-subjects factor ‘region’ resulted in a significant main effect of group F(1,16) = 8.490, p = .010, indicating that the CElo group showed overall significantly higher mean signal intensities in the regions active during task performance than the CEhi group. The main effect of area was significant F(16,256) = 4.420 p = .000, the interaction area*group was not significant F(16,256) = 1.532 p = .089.

T-tests for independent samples (two-tailed) showed that mean activation in bilateral retrosplenial cortex/cingulate regions was higher in CElo than in CEhi (t(16) = 2.589 p = .020), as was the mean activation in bilateral insula regions (t(16) = 2.990 p = .009) and in bilateral caudate (t(16) = 3.676 p = .002).

Statistically significant differences between the mean signal intensities in the CEhi and CElo groups were also found in the following regions: right caudate body, bilateral insula BA 13, left middle frontal gyrus BA 6 and 8, left precuneus BA 7 and right cingulate gyrus BA 31. In all of these regions, CEhi subjects show significantly less activation than CElo subjects.

See Figure 4b for examples and Table 4 for a complete list of individual regions which show significant differences between mean signal intensities of CEhi and CElo groups, calculated in t-tests for independent samples.

**Table 4:** Brain areas in which CElo group activation is significantly higher than CEhi group activation during performance of the motor sequence task.

Brain Area (BA)	MNI coordinates	Cluster size	t-value	p-value
**Right cingulate gyrus (BA 31)**	20 −26 36	34	t(16) = 2.125	p = .049
**Left precuneus (BA 7)**	−24 −62 52	54	t(16) = 2.056	p = .030
**Right insula (BA 13)**	34 −20 24	15	t(16) = 2.280	p = .037
**Left insula (BA 13)**	−26 −32 16	15	t(16) = 2.543	p = .022
**Right caudate body**	22 −20 22	26	t(16) = 2.705	p = .016
**Right caudate body**	4 8 16	10	t(16) = 3.250	p = .006
**Right caudate body**	4 4 22	37	t(16) = 2.596	p = .012
**Left middle frontal gyrus (BA 8)**	−28 12 38	20	t(16) = 2.659	p = .021
**Left precentral gyrus (BA 6)**	−20 −18 50	12	t(16) = 3.536	p = .003
**Left middle frontal gyrus (BA 6)**	−22 −12 44	11	t(16) = 3.608	p = .004

## Discussion

### The native variability of cortical excitability is inversely related to the variability of brain activation intensity during a motor sequence task

In our study we find a systematic relation between the native level of cortical excitability and the BOLD activation level during execution of a motor task requiring either low variability (performance) or high variability (improvisation) of output. In both conditions, participants with low baseline cortical excitability show significantly higher activation levels in task-relevant regions than participants with high baseline cortical excitability, an effect that was found significant in about half of the task-relevant clusters of the two conditions. In the high variability task, significant differences are found in predominantly left hemispheric prefrontal and cingulate cortex, in the low variability task, they are located in bilateral insula, caudate and cingulate. In all other task-relevant regions of right hippocampus, right posterior cingulate regions, left precuneus and left caudate tail during the low variability task (performance) and bilateral cerebellum, left parietal lobule, right medial and superior frontal gyrus and right superior and middle temporal gyrus during the high variability task (improvisation) we find an identical pattern of differences between groups - however, in these regions the differences fail to reach a statistically significant level.

GABAergic influences might play a role in both cortical excitability and BOLD activation intensity. Intracortical inhibition in cortical excitability is assumed to be GABA-mediated [14], thus a high level of inhibition might reflect high GABAergic activity. A study investigating the relation of baseline GABA concentration as measured by MRS and the fMRI response in visual cortex [27] found that the signal variations of the BOLD contrast are linked to baseline GABA levels in a significant negative correlation, associating higher BOLD signal changes with lower GABA levels and vice versa. Results from a study investigating the effects of Zolpidem, a drug enhancing GABAergic inhibition, on visual response to a checkerboard pattern [28] point into the same direction, linking GABA-mediated inhibition and reduced BOLD responses. GABA receptors as the major inhibitory neurotransmitter receptors are found abundantly in the cortex. Provided that GABA levels measured in visual cortex correspond to a great extent to overall GABA levels in the brain, it can be considered likely that the found relationship between GABA levels and BOLD response holds for other areas of the brain too.

In contrast to the findings described above, our results associate higher BOLD responses with those participants who according to their low level of cortical excitability should exhibit high GABAergic activity – i.e. the CElo group, and lower BOLD responses with those participants who according to their cortical excitability should have lower GABAergic activity – the CEhi group.

Assumed that in CElo participants the overall equilibrium of facilitation and inhibition is shifted towards inhibition, i.e. high overall GABA levels, they probably have to overcome this basic GABA influence by increased activation of excitatory systems during task performance, an effort that reflects in their higher BOLD response. Thus it is conceivable that the high BOLD response in these participants during the complex task might be a compensatory mechanism to overcome the high default GABA activity which otherwise might disturb successful performance of the task.

However, we cannot determine whether the increased amount of oxygen delivered to task-relevant regions on which the BOLD response is based predominantly serves to fuel excitatory glutamatergic connections or inhibitory GABAergic connections. Glutamatergic influences might also play an important role in both cortical excitability and the BOLD response. Intracortical facilitation is assumed to be mediated by glutamatergic systems - it can be reduced by the glutamate antagonist riluzole [15], [29], [30], and enhanced by the NMDA-antagonist ketamine, supposedly via an increase of glutamatergic transmission at non-NMDA receptors [31]. Thus low levels of facilitation might hint at reduced glutamatergic activity. Magnetic resonance spectroscopy as well as neurochemical and neurophysiological studies with fMRI suggest that the BOLD response from the cerebral cortex is closely linked to neurotransmitter release and energetic demand of glutamatergic neurons [32]. Correspondingly, animal research demonstrated that both NMDA and AMPA-antagonists decreased the BOLD response in rat somatosensory cortex to tactile forepaw stimulation [33].

Thus low glutamatergic activity is probably associated with a reduced BOLD response, suggesting in our case lower glutamatergic activation in the CEhi group than in the CElo group, which probably exhibits higher glutamatergic activation.

Taken together, our findings suggest a compensatory mechanism - the energy consumption required for adequate task performance might be higher in CElo subjects to overcome their high inhibition and boost their low facilitation, causing a higher BOLD activation than in CEhi subjects.

### Cortical excitability remains unchanged after learning and training of the motor sequence task

Contrary to our assumption in our first hypothesis, we did not find an overall increase in cortical excitability of motor cortex after learning and performance of a complex motor task. While inhibitory cortical excitability remained statistically unchanged in both groups, facilitatory cortical excitability experienced a significant decrease only in the CEhi group.

Some previous studies found overall increases in cortical excitability after learning of a sensory or motor task (e.g. [3], [5], [6], [7]). Differences in characteristics of the experimental design and task, in the method used to determine cortical excitability, as well as in measuring period/time may account for the diverging findings of our present experiment. The study by Muellbacher et al. [7], who observed an increase in the MEP amplitudes after learning, employed a non-sequence motor task, i.e. a ballistic contraction task, and a different measure for motor excitability - the resting motor threshold as well as MEPs evoked by only single pulse TMS. The studies by Abbruzzese et al. [3] and Koeneke et al. [6] which each found an increase in cortical excitability after learning too, also used only single pulse MEPs to establish the level of cortical excitability. The excitability thus determined was presumably measured during the performance of sequential movements; therefore these findings do not yield any information about post-learning changes of cortical excitability. Cirillo et al. [5] used a complex visuomotor task and paired pulses to determine cortical excitability, which they found increased after training.

In contrast to these studies, for determination of cortical excitability we used the ratio between responses to single pulses and double pulses. Several other studies that employed, like us, a ratio method of calculating cortical paired-pulse excitability [8], [34] also failed to find a change in cortical excitability in motor cortex after practicing of simple motor tasks.

It is thus possible that differences in the method of analyzing cortical excitability might account for the diverging findings. A major advantage of using a ratio method like that used by us is that it accounts for potential unspecific changes in overall excitability that might occur after training, thus this method should deliver more reliable information on changes in facilitation and inhibition.

Task difficulty does not appear to have a major role in observed increases or non-increases of cortical excitability. Both simple [6], [7], [34] or complex [3], [5] tasks could either cause or fail to cause an increase in cortical excitability, even though evidence of M1 being more involved in performance of complex than of simple tasks [35] might suggest that complex tasks would rather cause learning-dependent changes.

Moreover, the location of stimulation does not appear to be a relevant factor in whether CE increases after learning. We performed the TMS stimulation with a circular coil in a fixed location, positioned centrally over Cz, while other studies searched for an optimal hot spot for stimulation. (e.g. Ziemann et al. [8] stimulated at an “optimal site for eliciting MEPs in the left biceps”; Abbruzzese et al. [3] “positioned (the stimulation) over the left motor cortex to optimize the amplitude of the EMG responses”; Smyth et al. [34] located “the site eliciting the largest MEP in response to a moderately suprathreshold” stimulation, using a circular coil; Cirillo et al. [5] placed the coil in an “optimal scalp position over the left hemisphere to elicit a MEP in the relaxed right FDI muscle”; Koeneke et al. [6] applied “focal TMS (…) to the contralateral hand area of the motor cortex in order to determine the optimal scalp position for consistently eliciting motor evoked potentials (MEPs) of maximal amplitude in the target muscle”).

On the other hand, a first learning that might have changed cortical excitability might have occurred already before our measurements. In order to not have participants start from scratch during the fMRI measurement, we encouraged them to familiarize themselves with the keyboard layout and the motor sequence well ahead of the fMRI session. Those participants that actually did so were recognizable by their finishing the training session considerably faster than the others.

To check for this possibility of pre-experiment learning that might have escaped us, we first performed a median split of the complete group based on the duration of their training session. If early learning in some participants accounted for the lack of an overall change in group cortical excitability, we should see such a change nevertheless in those participants that did not familiarize themselves with the motor sequence in advance (slow learners), in contrast to those who learned early (fast learners). However, neither in fast nor in slow learners we found significant differences of cortical excitability measures between pre and post learning. Nor did we find significant between-group differences of these measures. The lack of change in cortical excitability pre and post training thus is presumably not attributable to pre-experiment learning.

### Variability of cortical excitability is not directly related to “success” parameters in motor sequence learning, such as high correctness and high levels of improvisation

Contradictory to our second hypothesis, the results show that having native low or high cortical excitability does not directly influence task performance, there are no significant group differences between CEhi and CElo groups with regard to percentage correct responses or improvisation level. This independence of performance of the differential levels of cortical excitability and activation in task-relevant regions suggest that there is no simple relation between performance and amount of activation or excitability.

### Activated brain regions in performance and improvisation

During improvisation of the motor sequence task we find significant activation in prefrontal, cingulate and temporal/parietal regions, in particular in bilateral orbitofrontal cortex, BA 11 and 47, right medial frontal gyrus BA 10 and left middle frontal gyrus BA 9, in bilateral cingulate gyrus BA 32 and left BA 24, in left BA 40 and in bilateral middle temporal gyrus BA 21 and right BA 22. These regions correspond partially to the activation pattern found during improvisation of musical themes, as demonstrated in several studies which reported activation in medial prefrontal cortex (BA 10), premotor/motor regions (BA 44,45,4,6), superior and middle temporal gyrus (BA 21,22), parietal cortex (BA 40, 7), anterior cingulate (BA 24,32) [22], [23], as well as middle frontal gyrus (BA 9) [24].

During reproduction of the prelearned motor sequence task, as compared to improvisation, we observed activation predominantly in temporoparietal, cingulate and subcortical regions, together with activated clusters in middle frontal gyrus. In particular, activation centered in left middle frontal BA 8 and superior frontal BA9, in the right retrosplenial cortex section [36], [37] of posterior cingulate BA 30/31, in right hippocampus, bilateral insula BA 13 and precuneus BA 7, left inferior parietal lobule BA 40 and bilateral caudate body as well as left caudate tail. Typical activation patterns during performance of a motor sequence task comprise motor cortex (BA 6), parietal cortex (BA 7,40), putamen, cerebellum, cingulate cortex (BA 23,24) [38]. Prefrontal cortex in general is assumed to participate in learning of new responses [38], with dorsal prefrontal cortex (BA 9, 46) and anterior cingulate (BA 23, 32) being particularly involved in attention to and awareness of sequence [38], [39]. Other regions found active during learning of new sequences more than in performance of prelearned sequences are caudate nucleus and globus pallidus [40], with caudate nucleus mediating either mental rehearsal or reinforcement of movements as a consequence of outcome. The posterior attentional system (parietal association cortex) directs attention towards extra personal space and sensory events [39]. The hippocampal system was found involved in both early and late stages of motor sequence learning [41], indicating a role for hippocampus in implicit motor sequence learning. Insular involvement in motor learning has been previously shown to occur during motor tasks involving both the upper and lower extremity [42]. Perfusion increases in retrosplenial cortex during performance of a motor sequence task were found in a study using 3T arterial spin labeling (ASL) [43].

Both for the performance and the improvisation condition, our results correspond largely to findings in the literature. While activation during performance corresponded to the pattern typically found in motor sequencing tasks associated with low variability of responding, activation during improvisation largely corresponded to results found in studies on musical improvisation, suggesting a common neural basis for tasks that process high variability of responding and require a modulation of previously learned material.

A number of the regions that were activated in improvisation and performance, respectively, did show only non-significant differences in activation between CEhi and CElo participants, even though the activation level was higher in the CElo group also in these areas. In the improvisation condition, these were located in right middle and inferior temporal gyrus (BA 21, 37), in left inferior parietal lobule (BA 40), as well as in cerebellum. In the performance condition, these were located in bilateral caudate tail, left caudate body, in a large parietal cluster of posterior cingulate, cingulate gyrus and precuneus, as well as in left precentral gyrus.

However, the ANOVA analyses in which we compared mean signal intensities between groups revealed an overall difference in activation between CEhi and CElo groups in both conditions. Further experiments will have to investigate what factors might determine significant or non-significant differences in activation levels of a given area between participants with high or low cortical excitability.

Several of the cortical regions showing differential activation in our CEhi and CElo groups have also been shown implicated in a modality-independent [44] fronto-parietal attentional network involved in top-down attentional control [45]–[47], i.e. anterior cingulate cortex, dorsolateral PFC and superior parietal regions [39], [48], with some studies also listing inferior frontal regions [44]. In improvisation, differential activation occurred in anterior cingulate, dorsolateral PFC, in performance, differences in activation were found in precuneus and inferior parietal lobule. However, since these activation differences did not translate into observable differences in behavior, with both groups showing equal levels in improvisation and correctness of performance, it is difficult to argue that they might be related to reduced or enhanced attention to the task.

### Conclusion

In this study we investigated whether the native level of cortical excitability influences potential learning-related changes of CE post-training, of brain activation and task performance.

The most prominent difference between persons with high or low cortical excitability was the differential intensity of their BOLD activation, with the CElo group showing a consistent pattern of higher mean signal intensity than the CEhi group across all task-relevant regions and in both task conditions. The finding may potentially reflect a compensatory mechanism in CElo subjects to overcome a high default GABA activity and boost low glutamatergic activity, which usually are associated to lower BOLD activation.

CE in this study, as in several previous ones, showed no significant learning-induced increase in the complete group or in participants with either low or high native CE, thus there is no support for a potential role for CE as a marker of cortical plasticity. Furthermore, the contradictory results from previous studies with regard to use-dependent increases in CE are presumably not related to the native level of CE. Further research is needed to determine the preconditions for increases in CE after motor learning and training. Moreover, the level of native CE did not influence performance and improvisation of a motor sequence task, suggesting that the basic level of motor cortex CE has no impact upon motor performance.
